# E-cigarette use and onset of first cigarette smoking among adolescents: An empirical test of the ‘common liability’ theory

**DOI:** 10.12688/f1000research.21377.3

**Published:** 2020-06-23

**Authors:** Hui G. Cheng, Edward G. Largo, Maria Gogova

**Affiliations:** 1 Regulatory Sciences, Altria Client Services, Richmond, VA, 23219, USA

**Keywords:** Adolescents, e-cigarette, cigarette smoking, common liability theory

## Abstract

**Background:** E-cigarettes have become the most commonly used tobacco products among youth in the United States (US) recently. It is not clear whether there is a causal relationship between e-cigarette use and the onset of cigarette smoking. The “common liability” theory postulates that the association between e-cigarette use and cigarette smoking can be attributed to a common risk construct of using tobacco products. This study aims to investigate the relationship between ever e-cigarette use and cigarette smoking onset in the US using a structural equation modeling approach guided by the “common liability” theory.

**Methods:** The study population is non-institutionalized civilian adolescents living in the US, sampled in the longitudinal Population Assessment of Tobacco and Health study. Information about tobacco product use was obtained via confidential self-report. A structural equation modeling approach was used to estimate the relationship between e-cigarette use at wave 1 and the onset of cigarette smoking at wave 2 after controlling for a latent construct representing a “common liability to use tobacco products.”

**Results: ** After controlling for a latent construct representing a “common liability to use tobacco products”, ever e-cigarette use does not predict the onset of cigarette smoking (β=0.13, 95% CI= -0.07, 0.32, p=0.204). The latent “common liability to use tobacco products” is a robust predictor for the onset of cigarette smoking (β=0.38; 95% CI=0.07, 0.69; p=0.015).

**Conclusions:** Findings from this study provide supportive evidence for the ‘common liability’ underlying observed associations between e-cigarette use and smoking onset.

## Introduction

The prevention of underage cigarette smoking is an essential component to curtail the substantial disease burden associated with cigarette smoking ^1^. The prevalence of underage cigarette smoking has been declining in the US over the past two decades ^2^. E-cigarettes (or e-vapor products) were introduced to the market as potentially reduced-harm alternatives to combustible cigarettes and have gained popularity since they became generally accessible around 2009 ^3, 4^. In the US, the prevalence of youth e-cigarette use increased sharply between 2011 and 2015 and, after a brief decline, increased again in 2018 and 2019 ^3, 4^. Multiple national surveys have shown that the majority of e-cigarette use is infrequent or experimental ^4– 6^. Despite this, the existence of exclusive e-cigarette users who do not use other tobacco products raises concerns about whether e-cigarettes may play a causal role in the uptake of cigarette use and, therefore, offset the decline in underage cigarette use.

A review of existing literature revealed some inconsistencies in evidence about the relationship between e-cigarette use and cigarette smoking. The largest body of literature comes from individual-level observational studies showing a consistent positive association linking e-cigarette use with a higher risk of cigarette smoking onset, even after adjusting for a range of variables known to be associated with cigarette smoking in a generalized linear regression framework ^7– 11^. Nonetheless, in the context of observational studies, it is almost impossible to control for an exhaustive list of confounders; the positive relationship can be attributed to unmeasured confounders associated with e-cigarette use, which would produce a spurious relationship between e-cigarette use and cigarette smoking without the presence of a causal relationship ^11– 13^. Propensity scoring is a statistical method to reduce biases due to confounding in observational studies to provide more robust evidence for causal inference ^14^. Recent studies using propensity scoring method have found either null or *inverse* association between e-cigarette use and smoking among youth ^15, 16^.

To infer causality, studies with an experimental design arguably provide the best evidence. Nonetheless, a randomized controlled trial of e-cigarette use among nonusers is not feasible ^12^. In this context, Vasiljevic and colleagues evaluated the impact of exposure to advertisements promoting e-cigarettes on appeal and susceptibility of tobacco smoking using an experimental design. They found no differences in appeal, susceptibility, or perceived harm of cigarette smoking between youth who were randomized to the exposure group and the control group ^17^. Despite the lack of direct behavioral measures, this study suggests that the exposure to e-cigarette advertisement had no acute effect on the measures related to cigarette smoking.

Ecological prevalence trends are inconsistent with the notion that e-vapor use increases cigarette smoking. At the country level, e-cigarettes emerged and gained popularity during the period when the sharpest decline in adolescent cigarette smoking was observed in the US and other countries ^5, 18, 19^. Total nicotine use have remained stable since the introduction of e-cigarettes ^20^. In an interrupted time series analysis, Dutra and Glantz found no difference in the rate of decline in ever (p=0.57) or current (p=0.23) cigarette smoking before and after 2009, when e-cigarette became available in the US market ^2^. In another study, Levy and colleagues found strong inverse associations between the prevalence of e-cigarette use and the prevalence of cigarette smoking among adolescents and young adults, which confluence with all US national surveys identified via a systematic literature search ^21^. Similar findings have been documented in a recent study by Foxon and Selya ^20^. Similarly, a few studies that compared youth cigarette smoking prevalence between states with and without regulations to prohibit sales of e-cigarettes to minors have produced inconsistent findings with two studies documenting lower youth cigarette smoking in states without an e-cigarette age restriction ^22, 23^, and one study documenting the opposite ^24^.

One potential explanation of the consistently observed association between e-cigarette use and cigarette smoking from individual observational studies is the “common liability” theory, which postulates that any observed relationships between e-cigarettes and combustible tobacco cigarettes is attributed to the “liability” to use tobacco products of the individual; once this “common liability” is controlled for, there is no causal relationship between e-cigarette use and cigarette smoking ^25– 28^. This ‘common liability’ that encompasses a range of genetic and behavioral factors may not be directly observable but gives rise to the use of various tobacco products (various psychoactive substances in the original theory). Several lines of evidence align with the “common liability” theory. First, the relationship is not unique to e-cigarettes. Various studies showed that the use of any type of tobacco products (e.g., cigar, hookah, etc.) and/or other substances (e.g., alcohol and cannabis) predicted cigarette smoking onset (e.g., 9, 10, 28, 29). Moreover, the risk of cigarette smoking onset increases with the number of types of tobacco product used ^9^. Findings from a recent study using a propensity scoring approach found supportive evidence that the frequently observed association between e-cigarette use and smoking is largely attributed to shared risk factors, using data from Monitoring the Future, a national school survey of 8 ^th^, 10 ^th^, and 12 ^th^ graders ^15^, Kim and Selya found that the association between e-cigarette use and current smoking became null after taking into account a range of variables encompassing demographic characteristics, use of other psychoactive substances, perceived peer cigarette use, risk-taking, socially maladaptive behaviors, attitude toward smoking, and parental education using propensity scoring ^15^. Another study using data from NYTS, another national school survey, found inverse relationships linking e-cigarette use and subsequent cigarette smoking after propensity score matching (i.e., evidence of ‘gateway out’) ^16^. Second, the relationships between cigarette smoking and e-cigarette use are often reciprocal. Numerous studies have documented that cigarette smoking is associated with higher risk of e-cigarette use ^10, 30– 32^. Indeed, a few studies have found that smoking is a stronger predictor for e-cigarette use compared to e-cigarette use as a predictor for cigarette smoking ^10, 16^. Third, e-cigarette use predicts the onset of a range of other substance use behaviors, not limited to cigarette smoking ^8, 29^. These lines of evidence support the notion that the relationship is not limited or specific to e-cigarettes, and common risk factors may underlie the use of various tobacco products ^33^.

Poorer inhibitory control and elevated impulsivity are phenotypes of externalizing problems, which has been linked to precocious use of various substances ^34– 37^. A study found similar levels of inhibitory control and impulsivity between youth e-cigarette users who never smoked a cigarette and youth smokers who never used an e-cigarette. Both groups showed poorer inhibitory control and elevated impulsivity compared to youth not using any tobacco products ^38^.

Given this background and guided by the ‘common liability’ theory, the aim of this study is to estimate the prospective relationship between e-cigarette ever use and the onset of cigarette smoking after controlling for a “common liability” to use tobacco in US adolescents using a structural equation modeling approach. In essence, we first created a unidimensional construct to represent the ‘liability’ to use tobacco products, and then regressed the onset of cigarette use on antecedent e-cigarette use taking into account the latent ‘liability’ to use tobacco products.

## Methods

### Study population and sample

The study population consisted of non-institutionalized civilian adolescents 12–17 years of age living in the US, sampled in the longitudinal Population Assessment of Tobacco and Health (PATH) study. A multi-stage sampling method was used to draw nationally representative samples after Institutional-Review-Board-approved parent consent and youth assent ^39^. In contrast to school surveys of adolescents, the PATH sample includes young people irrespective of school attendance, and its sampling frame includes college dormitories and children of active-duty military living in the US. More details about the PATH methodology is provided elsewhere ^39^.

In this study, PATH wave 1 (2013–2014) and wave 2 (2014–2015) data were used. Participation levels were 54% at the household level and 78% at the individual level for the youth survey at wave 1. Wave 2 follow-up rate was 88% for youth. Because the outcome in this study is the onset of cigarette smoking, the study sample consists of wave 1 never smokers who were followed up at wave 2. We did not include “aged-up” adolescents (i.e., those who became 18 at wave 2) to retain a sample relevant for underage smoking (n=9,045).

### Assessments

PATH confidential assessments were audio computer assisted self-interviews (ACASI), with standardized multi-item modules on use of various tobacco products, including cigarettes, e-cigarettes, cigars (including traditional cigars, cigarillos, and filtered cigars), smokeless tobacco, snus, hookah, pipe, dissolvable tobacco, bidis, and kretek. Survey questions about ever use of these tobacco products are typically in the format of “Have you ever smoked/used …, even one or two puffs/times?” In this study, the outcome is the onset of ever cigarette smoking at wave 2, which is defined as smoking cigarettes (even one or two puffs) for the first time between wave 1 and wave 2 assessments among adolescents who had never smoked cigarettes at wave 1. PATH also assessed lifetime history (i.e., ever use) of alcohol, cannabis, Ritalin ^®^ or Adderall ^®^, Painkillers/sedatives/tranquilizers, cocaine, stimulants, and other drugs, respectively.

Sex (male or female) and age categories (12–14 or 15–17 years of age at baseline) were included as covariates. (The PATH Public Use File only included a binary variable for age.) Information about these covariates is from survey items in the Demographics module. When these items are missing, information from the household screening roster is drawn. Other covariates include race/ethnicity (non-Hispanic White, non-Hispanic Black, Hispanics, and non-Hispanic others), availability of tobacco products in the household (yes/no), and a measure of novelty seeking (“agree” or “strongly agree” to the question “I like new and exciting experiences, even if I have to break the rules. Do you…”), participant’s self-rated health (excellent/non-excellent), harm perception of cigarettes (“a lot of harm” to the question “How much do you think people harm themselves when they smoke cigarettes”), and participant’s school performance (mostly A’s or A’s and B’s vs. others).

### Analysis approach

Guided by the “common liability” theory, we used a structural equation modeling approach to test whether there is a specific association between e-cigarette ever use at wave 1 and the onset of first cigarette smoking at wave 2 holding constant a latent construct for the “liability to use tobacco products.”

**Figure 1.**
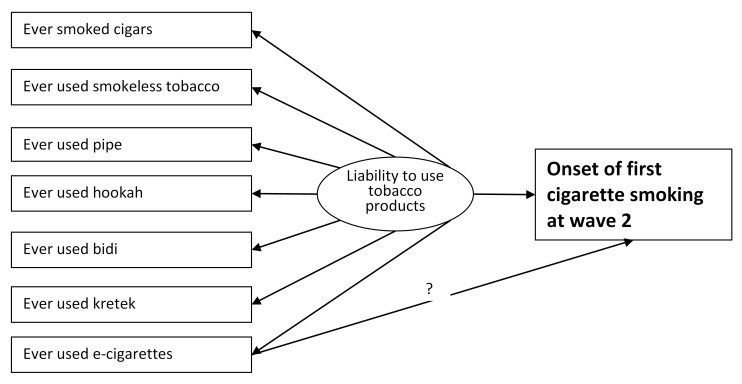
Depiction of a conceptual structural equation model to predict the onset of first cigarette smoking by e-cigarette use adjusting for a latent ‘liability to use tobacco’ construct.

Structural equation modeling is a collection of statistical techniques that allows simultaneous estimation of relationships of various independent and dependent variables. Compared to conventional single-equation linear models, SEM has several major advantages including a) the flexibility to incorporate various direct and indirect paths simultaneously based on theories or hypotheses, b) the ability to construct latent variables, c) evaluation of how well the specified model fits data via model fit indices, and d) minimization of measurement error via the construction of the latent construct. SEM has been used in tobacco research and yielded valuable insights ^40, 41^. It is particularly useful when a certain condition (e.g., depression, addiction, or, in this study, the liability to use tobacco products) cannot be directly observed but can be derived from a set of observable behaviors that often happen as a cluster because of the underlying condition. We consider SEM a suitable method to evaluate the common liability theory because the common liability is hypothesized as a latent construct that encompasses various genetic and environmental causes for tobacco use. 

in the first analysis steps, we built a latent construct for the common liability to use tobacco products using confirmatory factor analysis methods. The observed variables were lifetime ever use of specific tobacco products. All tobacco products assessed in PATH wave 1 were included to create the latent construct. Snuff and chewing tobacco, snus, and dissolvable tobacco products were combined to create a “smokeless tobacco products” variable due to the considerations that a) youth participants may not differentiate these oral tobacco products well, and b) low occurrence of dissolvable tobacco use (n=9) and high correlation between smokeless tobacco use and snus use (i.e., 69% of snus users had also used smokeless tobacco). All observed variables were treated as categorical variables. The variance of the latent construct was fixed to one in order to obtain factor loading and threshold estimates for each observed variable. After ensuring a good fit of the measurement model (as described in the next paragraph), we built the structural portion of the model to assess the relationship between e-cigarette ever use at wave 1 and the onset of first cigarette smoking at wave 2. Specifically, we drew a path from the latent construct to the onset of first cigarette smoking as well as a direct path from e-cigarette ever use to the onset of first cigarette smoking. Figure 1 provides a conceptual description of the model. If the direct path from e-cigarette to cigarette smoking is statistically robust, it provides evidence that e-cigarettes plays a role for cigarette smoking onset over and beyond the common liability to use tobacco products. If not, it supports the notion that the frequently observed association between e-cigarettes ever use and smoking is attributed to a common liability to use tobacco products.

A series of models were fit to explore the sensitivity of the main model. First, we expanded the measurement model to include other substances measured in PATH (i.e., alcohol, cannabis, prescription Ritalin and Adderall, cocaine/crack, stimulants, and other drugs (heroin, inhalants, solvents, and hallucinogens) in order to further test the model with a latent construct for the liability for use of psychoactive substances (as was originally proposed by the ‘common liability theory’). Second, we included additional sociodemographic and individual characteristics variables related to tobacco use in the model as covariates, including race/ethnicity, availability of tobacco products in the household, a measure of novelty seeking, participant’s self-rated health, harm perception of cigarettes, and participant’s school performance. In the last step of exploratory analyses, we repeated the main model with PATH wave 2 and 3 data as PATH wave 3 data became available soon after the initial analysis was conducted.

Several model fit indices were used to assess the goodness of fit of the measurement and the final structural models. These fit indices include root mean square of approximation (RMSEA) ^42^, comparative fit index (CFI) ^43^, and Tucker-Lewis index (TLI) . A RMSEA<0.08 and CFI/TLI > 0.90 are considered as indications of reasonably good model fit ^44, 45^.

Analysis weights were used to adjust for selection probability, nonresponse patterns, possible deficiencies in the sampling frame, and attrition. The PATH User Guide provides details about the calculation of weights ^39^. A robust weighted least square mean and variance (WLSMV) adjusted estimator, which uses a full weight matrix, was used to accommodate categorical variables and complex survey design. For all tobacco use variables, there were <3% missing values. None of wave 1 never smokers had missing values in all tobacco use variables, so we are able to include all never smokers in the main model. Balanced repeated replication method was used to generate standard errors and 95% confidence intervals (CI). Analyses were conducted using Stata 15.0 (StataCorp, College Station, Texas, USA) and Mplus 8.1 (Muthén & Muthén, Los Angeles, CA, USA).

## Results

Among 13,651 participants at wave 1, there were 11,792 never smokers, among whom 9,045 remained youth (12–17 years of age) and were followed up for wave 2 assessment. Of the 9,045 participants, 51% were males; 64% were 12–14 years of age at wave 1; and 54% were non-Hispanic whites. Table 1 presents the estimated ever use of tobacco products as well as factor loadings and thresholds from confirmatory factor analysis among youths who had never tried a cigarette at wave 1 (n=9,045). Among the sample of youth never smokers, e-cigarette was the most common tobacco product ever tried (3.7%), followed by hookah (2.5%), with bidi and kretek being the least common tobacco products ever tried (0.1%). The measurement model fits reasonably well (RMSEA=0.019, 90% CI=0.014, 0.024; CFI=0.963; TLI=0.945); all factor loadings were greater than 0.4 and statistically significant (p<0.001). Each individual’s factor score, a score reflecting the latent liability to use tobacco products, was calculated based on both the threshold and the loading of each item. Therefore, an individual who used bidi, kretek, or pipe (items with greater thresholds) would have a higher factor score compared to an individual who used e-cigarette or hookah only (items with lower thresholds). An individual who used multiple products would generally have a higher factor score than an individual who used a single product. This factor score represents an individual’s liability (unobserved) to use tobacco products.

**Table 1.** Estimated occurrence (%) of lifetime ever use of tobacco products, alcohol, cannabis, and other psychoactive drugs at wave 1, factor loadings, and thresholds from confirmatory factor analysis among never smokers. Data from Population Assessment of Tobacco and Health wave 1 and 2, 2013–2015 ^46^. (Unweighted n=9,045 12–17 Year Olds ^a^).

Ever use of	Weighted %	Tobacco-only measurement model		All psychoactive substance measurement model	
		Standardized factor loading ^d^	Unstandardized threshold	Standardized factor loading ^d^	Unstandardized threshold
E-cigarette	3.7	0.76	2.75	0.70	2.49
Cigar	1.3	0.71	3.16	0.72	3.16
Pipe	0.3	0.88	5.83	0.70	3.84
Hookah	2.5	0.62	2.51	0.68	2.68
Smokeless tobacco products ^b^	1.2	0.63	2.85	0.59	2.74
Bidi	0.1	0.45	3.63	0.46	3.87
Kretek	0.1	0.68	4.49	0.76	5.01
Alcohol	29.1			0.60	0.69
Cannabis	4.8			0.84	3.13
Prescription Ritalin and Adderall	1.1			0.53	2.69
Cocaine/crack	0.1			0.81	5.08
Stimulants	0.1			0.79	4.80
Other drugs ^c^	0.2			0.65	3.87
	Selected demographic characteristics				
Male	51.3				
12–14 years of age	63.7				
Non-Hispanic whites	53.8				

^a^Analytical sample consists of youths who had never smoked a puff of cigarette at wave 1 and followed up and remained youths at wave 2. ^b^ smokeless tobacco includes smokeless tobacco, snus, and dissolvable tobacco products. ^c^Other drugs include heroin, inhalants, solvents, and hallucinogens. ^d^Factor loadings are standardized based on the variances of the continuous latent variables as well as the variances of the outcome variables.

Of the 9,045 never cigarette smokers at wave 1 assessment, 338 adolescents smoked a cigarette for the first time between wave 1 and wave 2 assessments (weighted incidence= 3.8%). The structural equation model shown in Figure 1 fits data well (RMSEA=0.015, 90% CI=0.011, 0.020; CFI=0.969; TLI=0.954). The model shows that after accounting for the general liability to use tobacco products, the association between e-cigarette ever use at wave 1 and cigarette onset at wave 2 is not statistically significant (M1 in Table 2: β=0.13; 95% CI= -0.07, 0.32; p=0.204). The latent general liability variable is positively associated with wave 2 onset of ever cigarette smoking (β=0.38; 95% CI=0.07, 0.69; p=0.015). Because sex and age are the two most important exogenous variables that are related to tobacco use behaviors, they were introduced as covariates in the next step to assess any potential changes in estimates and statistical inference. Including sex and age as covariates introduced little change in estimates, and statistical inference remained the same (M2 in Table 2: β=0.16; 95% CI= -0.03, 0.35; p=0.095; for the e-cigarette to cigarette onset path; β=0.33; 95% CI= 0.04, 0.61; p=0.025; for the ‘liability to use tobacco’ latent construct to cigarette onset path).

**Table 2.** Estimated relationships linking e-cigarette use and common liability latent construct to onset of ever cigarette smoking from structural equation models. Data from Population Assessment of Tobacco and Health.

PATH waves	n ^a^	Model description ^b^	β (95% CI) for e-cigarette →smoking	β (95% CI) for common liability → smoking
1 & 2	9045	M1: Main model	0.13 (-0.07, 0.32)	0.38 (0.07, 0.69)
		M2: M1+ Sex & age	0.16 (-0.03, 0.35)	0.33 (0.04, 0.61)
		M3: M1 + Other psychoactive substances	0.13 (-0.002, 0.27)	0.44 (0.28, 0.61)
		M4: M3 + sex and age	0.14 (-0.002, 0.29)	0.43 (0.25, 0.60)
		M5: M1 + additional covariates	0.16 (-0.04, 0.35)	0.31 (0.01, 0.61)
		M6: M5 + additional covariates	0.16 (-0.04, 0.35)	0.30 (-0.02, 0.62)
2 & 3	8668	M1	0.15 (-0.06, 0.35)	0.34 (-0.03, 0.71)
		M3: M1 + Other psychoactive substances	0.13 (-0.03, 0.30)	0.42 (0.16, 0.68)

^a^Unweighted sample size. ^b^M1 is the model depicted in Figure 1. M5 includes the following covariates: race/ethnicity, availability of tobacco products in the household, and novelty seeking. M6 includes the following additional covariates: self-rated health, harm perception of cigarette smoking, and school performance.

In the first exploratory step, we expanded the measurement model to include other substances measured in PATH (see Table 1 for factor loading and thresholds). The expanded measurement and structural equation models both have good fit (RSMEA=0.015, 90% CI=0.012, 0.017; CFI=0.948, TLI=0.938 for the model without sex and age; RSMEA=0.016, 90% CI=0.015, 0.018; CFI=0.922, TLI=0.905 for the model out sex and age as covariates). Similar to results from the tobacco liability model shown above, the e-cigarette-specific path is not statistically significant (M3 in Table 2: β=0.13; 95% CI= -0.002, 0.27; p=0.053 without adjusting for sex and age; M4 in Table 2: β=0.14; 95% CI= -0.002, 0.29; p=0.054 with adjusting for sex and age), and the latent ‘liability’ construct is a robust predictor of cigarette smoking onset (β=0.44; 95% CI=0.28, 0.61; p<0.001 without adjusting for sex and age; β=0.43; 95% CI=0.25, 0.60; p<0.001 with adjusting for sex and age).

In the next exploratory step, we included two sets of sociodemographic and individual characteristics variables related to tobacco use in the model as covariates. First, we included race/ethnicity, availability of tobacco products in the household, and a measure of novelty seeking as covariates in the model. Inclusion of these covariates introduced little change in estimates, and statistical inference remained the same (M5 in Table 2). Next, we additionally included participant’s self-rated health, harm perception of cigarettes, and participant’s school performance. Again, inclusion of these additional covariates introduced little change in estimates (M6 in Table 2).

In final exploratory analysis steps, we repeated the main structural equation model with PATH wave 2 and 3 data as PATH wave 3 data became available soon after the initial analysis was conducted. Fit indices indicate that the model fit the data well (RSMEA=0.010, 90% CI=0.004, 0.015; CFI=0.992, TLI=0.988). Estimates are similar to those in the wave 1 and wave 2 model. That is, after accounting for the general liability to use tobacco products, the association between e-cigarette ever use at wave 2 and cigarette onset at wave 3 was not statistically significant (β=0.15; 95% CI= -0.06, 0.35; p=0.157). Possibly due to a reduced sample size of never smokers at wave 2 (n=8,668), the association leading from the latent general liability variable to wave 2 onset of ever cigarette smoking became statistically non-significant (β=0.34; 95% CI=-0.03, 0.71; p=0.074), although the point estimate is similar to that from the corresponding model using wave 1 and wave 2 data. As shown in Table 2, estimates for the e-cigarette-to-cigarette path is highly consistent across the series of models that we tested, which highlights the robustness of these structural equation models.

## Discussion

In this study, we found that the latent ‘common liability to use tobacco products’ construct is sufficient to explain later onset of smoking among US adolescents. In our exploratory analysis, we included various sets of covariates that have been shown to be associated with smoking onset. Estimates from these exploratory analyses are almost identical to the estimate from the main model, providing evidence that the model and the latent ‘common liability’ construct are robust.

### Interpretation of findings, implications, and future directions

A few hypotheses have been developed to provide theoretical grounds and underlying mechanisms for the relationship between e-cigarette use and subsequent cigarette smoking among youth. Here, we highlight three main theories as cited by the Academies of Sciences Engineering Medicine: the ‘diversion theory’, ‘catalyst theory’, and ‘common liability theory’ ^47^. In brief, the ‘diversion theory’ hypothesizes that e-cigarette deters tobacco cigarette use by diverting ‘high-risk’ individuals to e-cigarette from combustible cigarettes ^26, 48^. The ‘catalyst theory’ postulates that e-cigarette use increases the risk of combustible cigarette use by first attracting ‘low-risk’ individuals to e-cigarettes, as a reduced-harm product, and then increasing proclivity to try combustible tobacco cigarettes ^49^. The ‘common liability theory’ proposes that any observed relationships between e-cigarettes and combustible tobacco cigarettes is completely attributed to shared risk factors such as impulsivity and novelty-seeking (e.g., common liability to use tobacco products; ^25– 28^). If the ‘diversion theory’ is true, e-cigarette use should decrease the onset of cigarette smoking (or accelerate the declining trend of smoking); if the ‘catalyst theory’ is true, e-cigarette use should increase the onset of cigarette smoking; if the ‘common liability’ theory is true, a null relationship should be observed. Our findings provide supporting evidence for the ‘common liability’ theory for the US youth population as a whole. Recent studies using propensity scoring methods found supportive evidence that the observed association between e-cigarette use and current cigarette smoking was attributed to shared risk factors for tobacco use ^15, 16^. In this study, we found converging evidence using a different approach (i.e., SEM vs. propensity scoring), which accentuates the possibility that shared causes give rise to both e-cigarette use and cigarette smoking. Nonetheless, it is possible that different hypotheses apply to heterogeneous groups of individuals or contexts. For example, e-cigarettes may provide an alternative to cigarette smoking for some adolescents, whereas they may expose some other adolescents to a more smoking-prone environment. The onset of cigarette smoking is a complex interplay between micro-, meso-, and macro-level factors. Future studies with assessments of a breadth of these relevant factors and due attention to potential heterogeneities may provide more insights about the role of e-cigarette in smoking onset to guide targeted prevention and intervention efforts among youth. Applications of similar approaches in other cultures will help assess the reproducibility and consistency of the evidence, which is required for causal inference. It is noteworthy in this context that tobacco use behaviors in youth are often experimental and highly dependent on the availability of tobacco products ^6, 50– 52^, and an infrequent use pattern (1–2 days during the past 30 day) is more pronounced among exclusive e-cigarette users compared to youths who use other tobacco products concurrently ^51^. In this context, future studies incorporating the frequency of e-cigarette use and examining the relationship between e-cigarette use and the progression to more established cigarette smoking will provide useful insights about the e-cigarette-use-and-smoking relationship.

### Limitations and strengths

This study’s findings should be interpreted in light of the following limitations.

First, this study is observational in nature. Although we included multiple covariates in this study, unmeasured heterogeneity is possible (variables not accounted for by the latent ‘liability’ construct and other covariates not included in this study), and no definitive evidence for a causal relationship is warranted. Nonetheless, we observed high consistency in the estimates across models with different configurations, which supports the robustness of the model. Second, the assessment was based on self-report information. There is possible under-reporting of tobacco and other drug use due to socially desirable responding ^53^. The use of audio computer assisted self-interviews can help ameliorate this limitation. Measures for ever use of tobacco products have been shown to have good validity based on self-reported information ^54^. Third, the response level at the household screening is moderate. Nonetheless, it is comparable to other modern national household surveys in the US ^55^, and post-stratification was applied to bring the sample into balance with the US adolescent population.

Strengths of this study include a) the prospective design provides clear temporal relationship, and is less prone to differential recall as compared to cross-sectional surveys; b) compared to prevalence-based measures, newly incident cigarette use focuses on the onset process without any interference of the persistence process ^56^; c) by using nationally representative data, our results are generalizable to the general US adolescent population; and d) the use of audio computer assisted self-interviews and relatively low attrition in PATH enhances internal validity by reducing potential socially desirable responding and bias associated with attrition. In this study, we observed a much smaller point estimates for the e-cigarette-to-smoking relationship compared to estimates for the common-liability-to-smoking relationship. Together with the fairly large sample size of PATH (which grants reasonable statistical power), it suggests that the “common liability to tobacco use” is sufficient to explain the onset of cigarette smoking among adolescents.

Our approach is driven by the “common liability” theory. To the best of our knowledge, this is the first study to use a structural equation modeling approach to create a latent unidimensional ‘liability’ variable, which is consistent with the conceptualization of “liability”, which denotes “a latent (unobservable) quantitative trait that, when measured, ‘would give us a graded scale of the degree of affectedness or of normality’ ^27, 57^.”

Tobacco products use is the result of complex genetic-environment interplay. Under the “common liability” conceptualization, the sequence of tobacco product use is opportunistic and depends upon various environmental factors including accessibility, local policies, and social norms. Therefore, the threshold for each product may change from one culture to other. For example, in a cigar-prone culture, the threshold for cigar use may be lower than that for e-cigarettes. The latent ‘liability’ construct is capable of accommodating these types of environmental variations.

## Data availability

### Underlying data

National Addiction & HIV Data Archive Program: Population Assessment of Tobacco and Health (PATH) Study [United States] Public-Use Files (ICPSR 36498), https://doi.org/10.3886/ICPSR36498.v10 ^46^.

All data are publicly available. In order to download the data, users must first agree with the ICPSR Terms of Use, specific to each dataset, which can be viewed by clicking the download button. Data used in this study were downloaded on May 14, 2018.

## Acknowledgements

A previous version of this work is available from: https://dx.doi.org/10.2139/ssrn.3422321.
