# Supplementary material for: E-cigarette use and onset of first cigarette smoking among adolescents: An empirical test of the ‘common liability’ theory
Source: F1000Res. 2020 Jun 23;8:2099. Originally published 2019 Dec 13. [Version 3] doi: 10.12688/f1000research.21377.3 (PMC7366034; doi:10.12688/f1000research.21377.3)
Supplement: Peer review history

# Peer review: Reviewer response for version 3

**DOI:** 10.5256/f1000research.27508.r65344

**Published:** 2020-07-16

**Authors:** Arielle Selya

**Affiliations:** 1 Behavioral Sciences Group, Sanford Research, Sioux Falls, USA; 2 Pinney Associates, Inc., Pittsburgh, PA, USA

The authors have addressed all of my concerns.

Is the work clearly and accurately presented and does it cite the current literature?

Yes

If applicable, is the statistical analysis and its interpretation appropriate?

Yes

Are all the source data underlying the results available to ensure full reproducibility?

Yes

Is the study design appropriate and is the work technically sound?

Yes

Are the conclusions drawn adequately supported by the results?

Yes

Are sufficient details of methods and analysis provided to allow replication by others?

Yes

Reviewer Expertise:

Behavioral science, esp. nicotine use and dependence among adolescents

I confirm that I have read this submission and believe that I have an appropriate level of expertise to confirm that it is of an acceptable scientific standard.

# Peer review: Reviewer response for version 2

**DOI:** 10.5256/f1000research.26067.r63038

**Published:** 2020-06-02

**Authors:** Sandra I. Sulsky, Alexa Gallagher

**Affiliations:** 1 Ramboll, Amhurst, USA; 2 Ramboll US Corporation, Amherst, MA, USA

We were pleased to review the revised version of the Cheng *et al.* (2019) manuscript. The authors made several changes that strengthened and clarified the manuscript including a re-organization of the introduction with additional information on Kim *et al. *(2019) and a very recently published study by Shahab *et al.* (2020) and an explanation of the common liability theory. The methods section now includes more background information on SEM, which will be helpful to readers not familiar with this type of modeling. We were also pleased to see sensitivity analyses using PATH Wave 3 data as well as analyses including a broader set of covariates (age, sex, race/ethnicity, tobacco product availability in the household, novelty seeking, self-rated health, cigarette smoking harm perception, school performance). The authors provided detail about how missing information was handled. The results section now includes some basic demographic information, revisions to the tables, and additional results based on the new sensitivity analyses described in the methods.  

 We have a few minor suggestions: 

- On page 5, the first letter of the first complete paragraph needs to be capitalized.  

- Table 2 provides results for models M1 to M6 using PATH waves 1 and 2 data. The written results describe models M1 and M2 followed by M5 and M6. Models M3 and M4 are described last. Why not change the order of the models in the table so they are described in sequence in the results? 

- Please revisit the title of table 2. You present estimates for e-cigarette use and onset of ever cigarette smoking as well as estimates for the liability to use tobacco and the onset of ever cigarette smoking. The title only explains the e-cigarette piece. 

Is the work clearly and accurately presented and does it cite the current literature?

Partly

If applicable, is the statistical analysis and its interpretation appropriate?

Yes

Are all the source data underlying the results available to ensure full reproducibility?

Yes

Is the study design appropriate and is the work technically sound?

Yes

Are the conclusions drawn adequately supported by the results?

Yes

Are sufficient details of methods and analysis provided to allow replication by others?

Partly

Reviewer Expertise:

Epidemiology

We confirm that we have read this submission and believe that we have an appropriate level of expertise to confirm that it is of an acceptable scientific standard.

# Response

**Published:** 2020-06-18

**Authors:** Hui Cheng

**Affiliations:** Altria Client Services, USA

We are delighted to see that reviewers are happy about the revision. We appreciate reviewers’ constructive comments which have helped us improve the manuscript.

 We have made additional adjustments based on reviewers' comments. Specifically, we have changed the model labels, and we have changed the title of Table 2 to “ **Estimated relationships linking e-cigarette use and common liability latent construct to onset of ever cigarette smoking from structural equation models.  **Data from Population Assessment of Tobacco and Health survey”.

# Peer review: Reviewer response for version 2

**DOI:** 10.5256/f1000research.26067.r63037

**Published:** 2020-05-26

**Authors:** Arielle Selya

**Affiliations:** 1 Behavioral Sciences Group, Sanford Research, Sioux Falls, USA; 2 Pinney Associates, Inc., Pittsburgh, PA, USA

The authors have done an excellent job responding to my concerns. I agree that a latent factor for nicotine use would make sense as a single latent factor, and that additional covariates would add complications. However, the additional exploratory analyses which include such covariates show that the findings are robust. I have a few remaining comments:

- My biggest concern is that the authors should be careful not to conclude *no relationship* between e-cigarettes and later cigarette use. Using the significance testing here, that cannot be concluded. A couple specific points:
  - Such results should be worded as, e.g. “not significantly associated with” instead of “not associated with” (p. 6).
  - Though the Beta coefficient does not reach statistical significance, it does look like it’s trending towards a small but positive value (~0.13) based on most of the confidence interval being above 0. It is possible then, that with larger sample sizes, this could become significant.
  - Therefore it might be helpful to also phrase this as, “common liability is sufficient to explain later cigarette use” if this is backed up by your results.
  - In this vein, it might be valuable to do something like a nested model test of M1 vs. a model where the path from e-cigarettes to later cigarette use is removed; if the model fit does not substantially worsen, then this would support e-cigarettes not having any additional explanatory power in addition to that of the latent factor.
  - An inconsistency with respect to how p-values are treated: in one place, p=.054 is interpreted as “not statistically significant”; but in another place, p=.074 is interpreted as “marginally associated.” This highlights the importance of interpreting the effect sizes as well (e.g. Beta coefficients).
- It would be more appropriate to use “nicotine” rather than “tobacco” in most instances, as e-cigarettes contain nicotine but not tobacco.
- Another recent paper may be relevant in the literature review on common liability: Foxon & Selya, 2020, *Addiction* ^1^, which shows that total nicotine use trends (i.e. the use of any nicotine product) has not changed after the introduction of e-cigarettes.

Is the work clearly and accurately presented and does it cite the current literature?

Yes

If applicable, is the statistical analysis and its interpretation appropriate?

Yes

Are all the source data underlying the results available to ensure full reproducibility?

Yes

Is the study design appropriate and is the work technically sound?

Yes

Are the conclusions drawn adequately supported by the results?

Yes

Are sufficient details of methods and analysis provided to allow replication by others?

Yes

Reviewer Expertise:

Behavioral science, esp. nicotine use and dependence among adolescents

I confirm that I have read this submission and believe that I have an appropriate level of expertise to confirm that it is of an acceptable scientific standard, however I have significant reservations, as outlined above.

## References

[1] : Electronic cigarettes, nicotine use trends and use initiation ages among US adolescents from 1999 to 2018. *Addiction*.2020; 10.1111/add.15099 10.1111/add.15099 PMC760625432335976

# Response

**Published:** 2020-06-18

**Authors:** Hui Cheng

**Affiliations:** Altria Client Services, USA

We thank the reviewer for the positive response. We have amended the manuscript based on reviewer's comments.

 To address the reviewer's comment about non-significant relationships, we have made the following revisions to the manuscript: (1) we have changed description of null associations to “not statistically significant…”; (2) we have changed the first sentence in the Discussion section to “In this study, we found that the latent ‘common liability to use nicotine products’ construct is sufficient to explain later onset of smoking among US adolescents.”; (3) we have used consistent description for p-values and highlighted the importance of the beta coefficients (last paragraph of Results) and; (4) we have added the following language in the Discussion section “In this study, we observed a much smaller point estimates for the e-cigarette-to-smoking relationship compared to estimates for the common-liability-to-smoking relationship. Together with the fairly large sample size of PATH (which grants reasonable statistical power), it suggests that the “common liability to nicotine use” is sufficient to explain the onset of cigarette smoking among adolescents.”

 We agree with the reviewer that a test of nested models would help make the point that the e-cigarette-to-cigarette path does not substantially improve the model fit. Regrettably, the use of replicate weights (to properly calculate the variance) precludes such formal comparisons of model fit. Nonetheless, as we mentioned above, we have added language emphasizing the effect size estimates.

 With respect to the reviewer's comment about using 'nicotine' instead of 'tobacco,' we thank the reviewer for pointing it out. The point is well taken. Nonetheless, we decided to retain the term 'tobacco products' for the following reasons: (1) although e-cigarettes do not contain natural tobacco, they typically contain tobacco-derived nicotine; (2) the FDA has deemed and regulates e-cigarettes as tobacco products, (3) other nicotine products (e.g., nicotine replacement therapy or novel oral tobacco-derived nicotine products) are not included in this study. We hope these reasons are sufficient to justify our use of ‘tobacco’ products in this manuscript.

 We thank the reviewer to bring the recent paper to our attention. We have cited the paper in the Introduction section of the revised manuscript (reference #20).

# Peer review: Reviewer response for version 1

**DOI:** 10.5256/f1000research.23544.r57841

**Published:** 2020-01-28

**Authors:** Sandra I. Sulsky, Alexa Gallagher

**Affiliations:** 1 Ramboll, Amhurst, USA; 2 Ramboll US Corporation, Amherst, MA, USA

Cheng *et al.* (2019) examines the association between e-cigarette use and onset of cigarette smoking in youth between the ages of 12 and 17 years in the U.S. They utilize nationally representative and publicly available data from the PATH study. The research was guided by “common liability” theory, which “postulates that any observed relationship between e-cigarettes and combustible tobacco products is attributed to the liability to use tobacco products of the individual; once this common liability is controlled for, there is no causal relationship between e-cigarette use and cigarette smoking.” The authors report that they are the first study to use structural equation modeling to answer this question.

**Introduction**

- Cheng and colleagues ask an interesting and timely research question.  They include a series of recent publications in their background as they lay out their justification. The introduction, however, is rather long and somewhat unfocused.
- I would lead the discussion of the existing literature with the paragraph starting, “The largest body of literature comes from individual-level observational studies showing a consistent positive association…” The Vasiljevic study looking at exposure to e-cigarette advertisements does not seem directly relevant. Although I understand that it utilizes an experimental design, it could be cut to shorten the rather long introduction.
- The authors’ reference #26 should be discussed more fully in the introduction and discussion sections, as it identifies the components of common liability to use tobacco products and could shed light on the proper covariates to include in the SEM.
- The discussion of ecological studies is lengthy and could be shortened.
- Common liability theory should be introduced and described in the introduction, as it underlies the whole project. What is it, how is it measured, why is it appropriate for this project? 

**Methods**

- SEM is not that commonly used by epidemiologists. The paper would be more accessible if it included a brief introduction to SEM, including model selection, model evaluation, and how to interpret the results. The authors should explain why SEM is the right way to analyze a hypothesis based in common liability theory.
- The authors used data from Wave 1 (2013-2014) and Wave 2 (2014-2015) of the PATH study. Were PATH Wave 3 (2015-2016) data available and considered? If available but not included, why not?
- Are the questions the same in Wave 2 for those who are 18? If so, have you considered running a sensitivity model that includes the 18-year-olds? Have other authors made this same decision to exclude them? How many Wave 1 youth were excluded because they had aged up by Wave 2? It may make less sense for future studies to make this cut-off considering changes in the minimum legal age to purchase tobacco products.
    
- The authors ran two sets of models, one limited to tobacco products and a second, which they call the ‘expanded liability model’, that includes tobacco products and psychoactive substances. This expanded liability model is not introduced until the results section. It should be described in the methods.
- Sex and age were included as covariates. Were any of the other risk factors for smoking available in PATH, such as were identified in authors’ reference 26? If so, why not use those?
- Please provide additional information on missing information. The only mention of it is as it relates to the covariates of age and sex. The authors report that this information was obtained from the Demographics module, which if missing was taken from the household screening roster. Was there missing information for the questions related to tobacco products, alcohol, cannabis, and other psychoactive drugs. If so, provide numbers and describe how it was handled. If there was limited missing information, that could be noted as a strength. Also, was there evidence that the data were, or were not, missing systematically? This can be assessed by comparing the available information for respondents who answered the substance use questions vs. those who did not. 

**Results**

- Please provide the number of eligible participants at Wave 1 and Wave 2 and report numbers and reasons for any that were excluded.  This could be done in the form of a figure ending with the final number included in your analysis.
- Also, please include a baseline table that provides basic demographic information. This could also be described in text.
- The titles for tables 1 and 2 indicate that that data is from Wave 1 and Wave 2.  We are interested in ever use of the products listed in the table at Wave 1 and ever use of cigarettes at Wave 2. Please clarify what is provided in the table and update the table titles/descriptions.
- In fact, perhaps tables 1 and 2 could be combined to include demographic data from Wave 1; ever use of e-cigarette, cigar, pipe, hookah, SLT, bidi, alcohol, cannabis, prescription Ritalin and Adderall, cocaine/crack, stimulants, and other drugs at Wave 1; and ever cigarette use at Wave 3.
-  I’d suggest placing the model results described in the text in a table.

**Discussion**

- The authors conclude that “after accounting for the latent ‘common liability to use tobacco products’ construct, ever e-cigarette use does not predict the onset of smoking among US adolescents.” This statement is supported by the results provided, assuming that the variables considered by the authors reasonably captured the common liability they hypothesized exists. This point – whether or not the covariates included were sufficient – needs further explanation and justification.
- The authors identify several appropriate limitations and strengths. However, the limitations should address more explicitly the availability of data to describe smoking propensity, as defined in reference 26 or elsewhere. The authors identify lack of recall bias as a strength of the study, but the interval between waves in PATH is quite short, so recall bias is not a major concern.
- The discussion section should place the current results into context of what is already known about smoking initiation in youth. The authors note that their findings are in line with a study by Kim *et al.* (2019) and they should discuss this reference more fully. Several other observational studies are referenced in the introduction and further description of these observational studies might be useful here as well.

 Thank you for the opportunity to be a reviewer of this study. It is my recommendation that this paper be approved with reservations.

Is the work clearly and accurately presented and does it cite the current literature?

Partly

If applicable, is the statistical analysis and its interpretation appropriate?

Yes

Are all the source data underlying the results available to ensure full reproducibility?

Yes

Is the study design appropriate and is the work technically sound?

Yes

Are the conclusions drawn adequately supported by the results?

Yes

Are sufficient details of methods and analysis provided to allow replication by others?

Partly

Reviewer Expertise:

Epidemiology

We confirm that we have read this submission and believe that we have an appropriate level of expertise to confirm that it is of an acceptable scientific standard, however we have significant reservations, as outlined above.

# Response

**Published:** 2020-04-22

**Authors:** Hui Cheng

**Affiliations:** Altria Client Services, USA

Dr. Gallagher and Dr. Sulsky comments

 Cheng et al. (2019) examines the association between e-cigarette use and onset of cigarette smoking in youth between the ages of 12 and 17 years in the U.S. They utilize nationally representative and publicly available data from the PATH study. The research was guided by “common liability” theory, which “postulates that any observed relationship between e-cigarettes and combustible tobacco products is attributed to the liability to use tobacco products of the individual; once this common liability is controlled for, there is no causal relationship between e-cigarette use and cigarette smoking.” The authors report that they are the first study to use structural equation modeling to answer this question.

**1. Introduction**

 1.1 Cheng and colleagues ask an interesting and timely research question.  They include a series of recent publications in their background as they lay out their justification. The introduction, however, is rather long and somewhat unfocused.

Authors response: We thank the reviewer for recognizing the value of the research question. We have amended the introduction to make it more focused based on reviewers’ helpful comments as follows.

 1.2 I would lead the discussion of the existing literature with the paragraph starting, “The largest body of literature comes from individual-level observational studies showing a consistent positive association…” The Vasiljevic study looking at exposure to e-cigarette advertisements does not seem directly relevant. Although I understand that it utilizes an experimental design, it could be cut to shorten the rather long introduction.

Authors response: We have reorganized the discussion of existing literature in the revised manuscript. It now begins with observational studies, followed by the experimental study by Vasiljevic and colleagues, and then ecological evidence. The description of the Vasiljevic study has been shortened.

 1.3 The authors’ reference #26 should be discussed more fully in the introduction and discussion sections, as it identifies the components of common liability to use tobacco products and could shed light on the proper covariates to include in the SEM.

Authors response: We have added more details of reference #26 (now reference # 15) in the revised manuscript (the middle of the 5 ^th^ paragraph in the Introduction and the middle of the second paragraph in Discussion). During the revision, another study using similar method has been published. In this study, an inverse association was found between e-cigarette use and subsequent cigarette smoking using data from NYTS. We have added this study in the revised manuscript (ref. #16).

 1.4 The discussion of ecological studies is lengthy and could be shortened.

Authors response: We have shortened the discussion about ecological studies in the revised manuscript.

 1.5 Common liability theory should be introduced and described in the introduction, as it underlies the whole project. What is it, how is it measured, why is it appropriate for this project? 

Authors response: In the revised manuscript, we have added more details about the common liability theory (the beginning of the 5 ^th^ paragraph of the Introduction).

**2. Methods**

 2.1 SEM is not that commonly used by epidemiologists. The paper would be more accessible if it included a brief introduction to SEM, including model selection, model evaluation, and how to interpret the results. The authors should explain why SEM is the right way to analyze a hypothesis based in common liability theory.

Authors response: We have added a brief introduction of SEM and why we think SEM is appropriate for this analysis based on the common liability theory (2 ^nd^ paragraph under Analysis Approach).

 2.2 The authors used data from Wave 1 (2013-2014) and Wave 2 (2014-2015) of the PATH study. Were PATH Wave 3 (2015-2016) data available and considered? If available but not included, why not?

Authors response: PATH wave 3 data were not available when we started this project. During the course of the project, they became available. We did repeat the analysis using wave 2 and 3 data, and the results are similar. We have added estimates using wave 2 and 3 data in the Results section of the revised manuscript and in the new Table 3.

 Nonetheless, we consider wave 1 and 2 data our primary focus because it is what we set out to do. Also, PATH wave 1 is the true baseline cohort, which is a representative sample of youth. Wave 2 is a follow-up of the wave 1 cohort with ‘shadow’ youth (those younger than 12) sampled at wave 1. In addition, for longitudinal studies like PATH, attrition occurs at each follow-up. When using wave 2 and 3 data, attrition may play a larger role.

 2.3 Are the questions the same in Wave 2 for those who are 18? If so, have you considered running a sensitivity model that includes the 18-year-olds? Have other authors made this same decision to exclude them? How many Wave 1 youth were excluded because they had aged up by Wave 2? It may make less sense for future studies to make this cut-off considering changes in the minimum legal age to purchase tobacco products.

Authors response: We did discuss whether we should include aged-up youth in the analysis, and we decided not to because the focus of this study is on youth smoking onset. During wave 1 and 2 data collection (2013-2015), cigarettes were legal for 18-year-olds to purchase in most places. Risk profiles and mechanisms may differ for underage smoking from of-age smoking. We wanted to retain a focus on youth smoking onset and, therefore, did not include 18 year olds in the current study.

 2.4 The authors ran two sets of models, one limited to tobacco products and a second, which they call the ‘expanded liability model’, that includes tobacco products and psychoactive substances. This expanded liability model is not introduced until the results section. It should be described in the methods.

Authors response: We have added a paragraph in the Method section to describe a series of models to explore the sensitivity of the main model results, including the model with additional psychosocial covariates and the model with wave 2 and wave 3 data, as suggested by the reviewers (fourth paragraph in the Analysis section).

 2.5 Sex and age were included as covariates. Were any of the other risk factors for smoking available in PATH, such as were identified in authors’ reference 26? If so, why not use those?

Authors response: We have added additional covariates in the series of exploratory analysis described in the revised manuscript. Please see our response to reviewer #1’s comment #2 for a detailed explanation.

 2.6 Please provide additional information on missing information. The only mention of it is as it relates to the covariates of age and sex. The authors report that this information was obtained from the Demographics module, which if missing was taken from the household screening roster. Was there missing information for the questions related to tobacco products, alcohol, cannabis, and other psychoactive drugs. If so, provide numbers and describe how it was handled. If there was limited missing information, that could be noted as a strength. Also, was there evidence that the data were, or were not, missing systematically? This can be assessed by comparing the available information for respondents who answered the substance use questions vs. those who did not. 

Authors response: We have added a sentence in the revised manuscript to indicate that for all tobacco use variables, there were <3% missing values. In addition, we specified a robust weighted least square mean and variance adjusted estimator which uses a full weight matrix. Therefore, a participant is included in the model unless the participant has missing values in all indicator variables (i.e., tobacco use and substance use variables). None of wave 1 never smokers had missing values in all of these variables, so we are able to include all never smokers in our model. We have added this information in the last paragraph of the Methods section in the revised manuscript.

**3. Results**

 3.1 Please provide the number of eligible participants at Wave 1 and Wave 2 and report numbers and reasons for any that were excluded.  This could be done in the form of a figure ending with the final number included in your analysis.

Authors response: In the revised manuscript, we added a sentence about the number of participants included in the current study:

 Among 13,651 participants at wave 1, 11,792 were never smokers, among whom 9,045 remained youth (12-17 years of age) and were followed up for wave 2 assessment.

 The sampling procedure of PATH is described in detail in PATH User’s Guide (ref. #39)

 3.2 Also, please include a baseline table that provides basic demographic information. This could also be described in text.

Authors response: In the revised manuscript, we have added basic demographics of the sample in Table 1 and a sentence in the first paragraph of the Results section:

 Of the 9,045 participants, 51% were males; 64% were 12-14 year olds; and 54% were non-Hispanic whites.

 3.3 The titles for tables 1 and 2 indicate that that data is from Wave 1 and Wave 2.  We are interested in ever use of the products listed in the table at Wave 1 and ever use of cigarettes at Wave 2. Please clarify what is provided in the table and update the table titles/descriptions.

Authors response: We have adjusted the titles of Table 1 and Table 2 to reflect the estimates are among wave 1 never smokers.

 3.4 In fact, perhaps tables 1 and 2 could be combined to include demographic data from Wave 1; ever use of e-cigarette, cigar, pipe, hookah, SLT, bidi, alcohol, cannabis, prescription Ritalin and Adderall, cocaine/crack, stimulants, and other drugs at Wave 1; and ever cigarette use at Wave 3.

Authors response: In the revised manuscript, we have combined the previous Table 1 and 2 into one table (new Table 1).

 3.5 I’d suggest placing the model results described in the text in a table.

Authors response: We have added Table 2 which contains model results.

**4. Discussion**

 4.1 The authors conclude that “after accounting for the latent ‘common liability to use tobacco products’ construct, ever e-cigarette use does not predict the onset of smoking among US adolescents.” This statement is supported by the results provided, assuming that the variables considered by the authors reasonably captured the common liability they hypothesized exists. This point – whether or not the covariates included were sufficient – needs further explanation and justification.

Authors response: As we indicated above, we have added additional covariates in the model in our exploratory analyses, and the estimates were very similar to the one from the main model. We think these additional results suggest a certain degree of robustness of the latent ‘common liability’ construct and the main model overall. We have added this information and related discussion in the revised manuscript.

 Please also see our response to comment 2.5 and to reviewer #1’s comment #2 for more details.

 4.2 The authors identify several appropriate limitations and strengths. However, the limitations should address more explicitly the availability of data to describe smoking propensity, as defined in reference 26 or elsewhere. The authors identify lack of recall bias as a strength of the study, but the interval between waves in PATH is quite short, so recall bias is not a major concern.

Authors response: Regarding concerns about other covariates, we have conducted additional exploratory analyses to incorporate additional covariates related to smoking onset based on previous literature. Please also see our response to comment 2.5 and to reviewer #1’s comment #2.

 Regarding recall bias, we understand that the follow-up interval is only a year; however, there has been literature showing inconsistencies in the reporting of tobacco use as well as alcohol drinking behaviors in the youth population. In a cross-sectional setting, a smoker may be more likely to recall other tobacco use and vice versa because they can be correlated behaviors. Therefore, we think it is a noteworthy strength of the current study.

 4.3 The discussion section should place the current results into context of what is already known about smoking initiation in youth. The authors note that their findings are in line with a study by Kim *et al.* (2019) and they should discuss this reference more fully. Several other observational studies are referenced in the introduction and further description of these observational studies might be useful here as well.

Authors response: We have revised both the Introduction and Discussion to provide fuller details of previous studies and their relevance to the current study to help put findings from the current study into context.

 In closing, we thank all three reviewers for their helpful and constructive comments. We hope we have provided sufficient information and revision to make the work acceptable for publication.

# Peer review: Reviewer response for version 1

**DOI:** 10.5256/f1000research.23544.r57839

**Published:** 2020-01-08

**Authors:** Arielle Selya

**Affiliations:** 1 Behavioral Sciences Group, Sanford Research, Sioux Falls, USA; 2 Pinney Associates, Inc., Pittsburgh, PA, USA

This manuscript focuses on the controversial question of whether e-cigarettes causally lead to later cigarette smoking among adolescents, or whether the use of both conventional cigarettes and e-cigarettes are due to a common liability. The paper uses structural equation modeling to model the pre-existing likelihood of e-cigarette use as a latent factor, finds that e-cigarettes do *not* have a causal effect on later conventional smoking over and above the latent factor. This paper uses an innovative method to address a complicated statistical question. The paper is well-written, and the implications are important.

 Major comments:

- The introduction has an excellent literature review that makes a convincing case for the common liability hypothesis, even using papers that at face value support the “gateway” hypothesis of e-cigarettes but identifying interesting counterpoints in the analyses of these papers.
- My main concern is about the choice to include only substance use variables in the latent factor representing liability to use tobacco products. Propensity to use tobacco products includes a variety of other factors as well (socioeconomic, personality characteristics, risk perception, peer group and family substance use, mental health, etc). Are these other variables available in the PATH study, and if so what was the rationale to restrict only to behavioral tobacco use measures?  What would the results look like if other covariates were also included?
- I would recommend a re-organization of the Discussion, to discuss interpretation of findings, implications, and relation to existing literature first, followed by limitations and strengths.

Minor comments:

- I think a brief description of “standardized threshold” (which appears first in Table 1) would be helpful.
- On p. 6, last paragraph, why are exact values not reported for the fit measures (“RMSEA <0.02, CFI and TLI > 0.90”) as they are elsewhere?
- What does the acronym ACASI mean (p.7)?

Is the work clearly and accurately presented and does it cite the current literature?

Yes

If applicable, is the statistical analysis and its interpretation appropriate?

Yes

Are all the source data underlying the results available to ensure full reproducibility?

Yes

Is the study design appropriate and is the work technically sound?

Yes

Are the conclusions drawn adequately supported by the results?

Yes

Are sufficient details of methods and analysis provided to allow replication by others?

Yes

Reviewer Expertise:

Behavioral science, esp. nicotine use and dependence among adolescents

I confirm that I have read this submission and believe that I have an appropriate level of expertise to confirm that it is of an acceptable scientific standard, however I have significant reservations, as outlined above.

# Response

**Published:** 2020-04-22

**Authors:** Hui Cheng

**Affiliations:** Altria Client Services, USA

Dr. Selya’s comments

General comments:

- This manuscript focuses on the controversial question of whether e-cigarettes causally lead to later cigarette smoking among adolescents, or whether the use of both conventional cigarettes and e-cigarettes are due to a common liability. The paper uses structural equation modeling to model the pre-existing likelihood of e-cigarette use as a latent factor, finds that e-cigarettes do *not* have a causal effect on later conventional smoking over and above the latent factor. This paper uses an innovative method to address a complicated statistical question. The paper is well-written, and the implications are important. Authors response: We thank the reviewer for recognizing the significance and novelty of the work and the helpful comments. We have revised the original manuscript based on these comments as detailed below.

Major comments:

 

- 1. The introduction has an excellent literature review that makes a convincing case for the common liability hypothesis, even using papers that at face value support the “gateway” hypothesis of e-cigarettes but identifying interesting counterpoints in the analyses of these papers.

Authors response: We thank the reviewer for recognizing the value of the research question.

-  2. My main concern is about the choice to include only substance use variables in the latent factor representing liability to use tobacco products. Propensity to use tobacco products includes a variety of other factors as well (socioeconomic, personality characteristics, risk perception, peer group and family substance use, mental health, etc). Are these other variables available in the PATH study, and if so what was the rationale to restrict only to behavioral tobacco use measures?  What would the results look like if other covariates were also included?

Authors response: We agree with the reviewer that the propensity to use tobacco products is multifaceted, including important factors mentioned by the reviewers.

In this study, we were guided by the ‘common-liability’ theory and hypothesized that the use of various tobacco products is driven by an unobserved latent liability to use tobacco products. That is, a single-dimension latent variable (i.e., the liability to use tobacco products) gives rise to the use of different tobacco products. This latent liability to use tobacco products likely varies by socioeconomic, personality characteristics, risk perception, peer influence, and familial history of substance use, mental health etc., which can be precedents, consequences, or both (feedback loops) of tobacco use. That is, our expectation is that the latent variable, which represents an individual’s liability to use tobacco products, is correlated with these other variables. Practically, one complexity to include various other factors in the structural equation framework is that these variables are not necessarily manifestations of a single dimension, which complicates the structure of the measurement model. Due to the above theoretical and practical reasons, we chose to take a simpler theory-driven approach to define the latent construct by tobacco use behaviors and only included sex and age as covariates because they are exogenous to the liability-and-smoking-onset relationship. In an exploratory step, we further expanded the model to include other substances because it has been shown that adolescent substance use is highly opportunistic and tobacco use is highly correlated with other substance use among adolescents in the original submission.

That said, the reviewer’s concern is well taken. We have expanded the model to include aforementioned variables as covariates to the extent that they were assessed in PATH wave 1 survey. Specifically, we took a two-step approach to include additional covariates. First, we added race/ethnicity (non-Hispanic White, non-Hispanic Black, Hispanics, and non-Hispanic others), availability of tobacco products in the household (yes/no), and a measure of novelty seeking (“agree” or “strongly agree” to the question “I like new and exciting experiences, even if I have to break the rules. Do you…”). In the second step, we additionally included participant’s self-rated health (excellent/non-excellent), harm perception of cigarettes (“a lot of harm” to the question “How much do you think people harm themselves when they smoke cigarettes”), and participant’s grade (mostly A’s or A’s and B’s vs. others) because these variables are more likely to have feedback loops with tobacco use. These covariates were modeled to correlate with the latent liability construct and to predict the onset of cigarette smoking at wave 2.

The inclusion of these variables induced little change in estimates, although the latent-liability-to-smoking-onset path became marginally significant statistically, possibly due to the loss of precision associated with the additional covariates. Nonetheless, e-cigarette did not predict smoking onset in either model (Additional model 1: β=0.16; 95% CI= -0.04, 0.35; p=0.109; Additional model 2: β=0.16; 95% CI= -0.03, 0.35; p=0.102), and the latent liability of tobacco use predicted smoking onset in the first additional model (β=0.31; 95% CI= 0.01, 0.61; p=0.047) as well as in additional model 2 (β=0.31; 95% CI= 0.01, 0.61; p=0.047).

We observed little change in estimates for the e-cigarette-to-smoking relationship as well as the liability-to-smoking relationship across the four models (no covariates, sex-and-age as covariates, additional model 1, and additional model 2 described above), which suggests that the model is robust to variations of covariates.

In the revised manuscript, we have added this additional information in the Results section and revised the Discussion section accordingly.

 

- 3. I would recommend a re-organization of the Discussion, to discuss interpretation of findings, implications, and relation to existing literature first, followed by limitations and strengths.

Authors response: We thank the reviewer for the suggestion. We have moved the ‘Limitation and strengths’ section to the end of the manuscript.

**Minor comments:**

 

- 1. I think a brief description of “standardized threshold” (which appears first in Table 1) would be helpful.

Authors response: We thank the reviewer for bringing it to our attention. After further consideration, we think the unstandardized thresholds are more meaningful than the standardized threshold. In the revised version, we have changed the standardized thresholds to unstandardized thresholds, which represents the ‘difficulty parameter’ of items. We have also added the following footnote for “standardized loading” in Table 1:

“Factor loadings are standardized based on the variances of the continuous latent variables as well as the variances of the outcome variables.”

 

- 2. On p. 6, last paragraph, why are exact values not reported for the fit measures (“RMSEA <0.02, CFI and TLI > 0.90”) as they are elsewhere?

Authors response: In the revised version, we have added exact values for these fit indices in the revised version as follows,

“RSMEA=0.015, 90% CI=0.012, 0.017; CFI=0.948, TLI=0.938 for the model without sex and age; RSMEA=0.016, 90% CI=0.015, 0.018; CFI=0.922, TLI=0.905 for the model out sex and age as covariates”

We did not report the exact values in the previous version because we considered the expanded model a post-estimation exploratory step and the results are similar to those of the main model. 

 

- 3. What does the acronym ACASI mean (p.7)?

Authors response: ACASI stands for audio computer assisted self-interviews. It was defined at first appearance under “Assessment.” Since it only appeared three times in the manuscript, we have replaced the acronyms with its full name in the revised version.
